# Association Between Awareness of Limiting Food Intake and All-cause Mortality: A Cohort Study in Japan

**DOI:** 10.2188/jea.JE20220354

**Published:** 2024-06-05

**Authors:** Daisaku Nishimoto, Rie Ibusuki, Ippei Shimoshikiryo, Kenichi Shibuya, Shiroh Tanoue, Chihaya Koriyama, Toshiro Takezaki, Isao Oze, Hidemi Ito, Asahi Hishida, Takashi Tamura, Yasufumi Kato, Yudai Tamada, Yuichiro Nishida, Chisato Shimanoe, Sadao Suzuki, Takeshi Nishiyama, Etsuko Ozaki, Satomi Tomida, Kiyonori Kuriki, Naoko Miyagawa, Keiko Kondo, Kokichi Arisawa, Takeshi Watanabe, Hiroaki Ikezaki, Jun Otonari, Kenji Wakai, Keitaro Matsuo

**Affiliations:** 1 Department of Epidemiology and Preventive Medicine, Kagoshima University Graduate School of Medical and Dental Sciences, Kagoshima, Japan; 2 School of Health Sciences, Faculty of Medicine, Kagoshima University, Kagoshima, Japan; 3 Department of Community-Based Medicine, Kagoshima University Graduate School of Medical and Dental Sciences, Kagoshima, Japan; 4 Environmental Epidemiology Section, Health and Environmental Risk Division, National Institute for Environmental Studies, Tsukuba, Japan; 5 Kagoshima Prefectural Oshima Hospital, Amami, Japan; 6 Community Medicine Support Center, Kagoshima University Hospital, Kagoshima, Japan; 7 Division of Cancer Epidemiology and Prevention, Aichi Cancer Center, Nagoya, Japan; 8 Division of Cancer Information and Control, Aichi Cancer Center Research Institute, Nagoya, Japan; 9 Division of Descriptive Cancer Epidemiology, Nagoya University Graduate School of Medicine, Nagoya, Japan; 10 Department of Preventive Medicine, Nagoya University Graduate School of Medicine, Nagoya, Japan; 11 Department of Preventive Medicine, Faculty of Medicine, Saga University, Saga, Japan; 12 Department of Pharmacy, Saga University Hospital, Saga, Japan; 13 Department of Public Health, Nagoya City University Graduate School of Medical Sciences, Nagoya, Japan; 14 Department of Epidemiology for Community Health and Medicine, Kyoto Prefectural University of Medicine, Kyoto, Japan; 15 Department of Endocrine and Breast Surgery, Kyoto Prefectural University of Medicine, Kyoto, Japan; 16 Laboratory of Public Health, Division of Nutritional Sciences, School of Food and Nutritional Sciences, University of Shizuoka, Shizuoka, Japan; 17 Department of Preventive Medicine and Public Health, Keio University School of Medicine, Tokyo, Japan; 18 Department of Public Health, Shiga University of Medical Science, Otsu, Japan; 19 NCD Epidemiology Research Center, Shiga University of Medical Science, Otsu, Japan; 20 Department of Preventive Medicine, Tokushima University Graduate School of Biomedical Sciences, Tokushima, Japan; 21 Department of General Internal Medicine, Kyushu University Hospital, Fukuoka, Japan; 22 Department of Comprehensive General Internal Medicine, Kyushu University Faculty of Medical Sciences, Fukuoka, Japan; 23 Department of Psychosomatic Medicine, Graduate School of Medical Sciences, Kyushu University Hospital, Fukuoka, Japan; 24 Department of Cancer Epidemiology, Nagoya University Graduate School of Medicine, Nagoya, Japan

**Keywords:** awareness of limiting food intake, all-cause mortality, cohort study

## Abstract

**Background:**

Improving diets requires an awareness of the need to limit foods for which excessive consumption is a health problem. Since there are limited reports on the link between this awareness and mortality risk, we examined the association between awareness of limiting food intake (energy, fat, and sweets) and all-cause mortality in a Japanese cohort study.

**Methods:**

Participants comprised 58,772 residents (27,294 men; 31,478 women) aged 35–69 years who completed baseline surveys of the Japan Multi-Institutional Collaborative Cohort Study from 2004 to 2014. Hazard ratios (HRs) for all-cause mortality and 95% confidence intervals (CIs) were estimated by sex using a Cox proportional hazard model, with adjustment for related factors. Mediation analysis with fat intake as a mediator was also conducted.

**Results:**

The mean follow-up period was 11 years, and 2,516 people died. Estimated energy and fat intakes according to the Food Frequency Questionnaire were lower in those with awareness of limiting food intake than in those without this awareness. Women with awareness of limiting fat intake showed a significant decrease in mortality risk (HR 0.73; 95% CI, 0.55–0.94). Mediation analysis revealed that this association was due to the direct effect of the awareness of limiting fat intake and that the total effect was not mediated by actual fat intake. Awareness of limiting energy or sweets intake was not related to mortality risk reduction.

**Conclusion:**

Awareness of limiting food intake had a limited effect on reducing all-cause mortality risk.

## INTRODUCTION

In 2017, an estimated 11 million people died worldwide due to noncommunicable diseases; 29% of these deaths were due to diet, in which unbalanced intake of fat and sugar-containing beverages played a major role.^1^ Furthermore, overeating is one of the causes of noncommunicable diseases, and an excessive intake of energy, fat, and sweets is associated with mortality risk.^2^^–^^4^ Therefore, the prevention of overeating and relevant dietary behavior changes are important.

Awareness is the first stage of behavioral changes. Prochaska et al proposed a behavioral change stage model, wherein awareness transforms into behaviors (Transtheoretical Model), with five stages in the health transformation process: precontemplation, contemplation, preparation, action, and maintenance.^5^ The Transtheoretical Model has been used as a framework in interventions for smoking cessation,^6^^,^^7^ as well as diet^8^^,^^9^ and exercise.^10^^,^^11^ Furthermore, numerous efforts have focused on increasing awareness of limiting foods for which overconsumption is a health problem. For example, in the United States, calorie labeling has been stipulated by law since 2018, with an estimated savings of $260 million over a 6-year period (from 2018 to 2023) compared to conventional medical expenses.^12^ Furthermore, a meta-analysis reported energy and fat intake as negatively associated with calorie and nutrient content labeling.^13^ Similar labeling has also resulted in a reduction in the purchase of sugar-containing beverages,^14^ and, in some subgroups, has resulted in reduced energy intake, medical costs, and body weight.^15^ Awareness of food intake restrictions may help prevent overeating.

Awareness of limiting food intake will mediate food intake and be associated with death as an independent factor, similar to noncommunicable diseases, exercise,^16^ and smoking.^17^ Although studies on the association between awareness of limiting foods for which overconsumption is a health problem and dietary behaviors have been reported, studies on the association between such awareness and the risk of death, as well as on factors that mediate this causation, is limited. Therefore, we evaluated the association between awareness of limiting intake of energy, fat, and sweets and all-cause mortality in a Japanese cohort. Our hypothesis is that individuals with awareness of limiting food intake (energy, fat, and sweets) are less likely to overeat and consequently have lower mortality.

## METHODS

### Participants

This study used data from the Japan Multi-Institutional Collaborative Cohort (J-MICC) Study. Details of the J-MICC study are available elsewhere.^18^^–^^20^ Briefly, the J-MICC study is a molecular epidemiological study aimed at preventing lifestyle-related diseases in Japanese people. In this study, residents in the community, health checkup examinees, and first-visit patients at a cancer hospital were recruited. Baseline surveys were conducted from 2004 to 2014 and were completed by 92,525 Japanese adults aged 35–69 years (dataset 20220809). The target regions were Chiba, Shizuoka, Aichi, Mie, Shiga, Kyoto, Tokushima, Fukuoka, Saga, Nagasaki, Kagoshima, and Okinawa. Those who submitted written informed consent were selected as research participants.

Of the 92,525 participants in the J-MICC Study, 59,682 had available data on awareness of limiting intake of energy, fat, and sweets, food intake, blood pressure, serum lipid levels, fasting blood glucose or HbA1c levels, and history of treatment for hypertension, dyslipidemia, or diabetes. As a result, all participants from Chiba were excluded. We further excluded those with no follow-up data (*N* = 38), those who died within 1 year of follow-up (*N* = 76), and those with daily energy intake <1,000 kcal or >4,000 kcal (*N* = 796). Finally, 58,772 people (27,294 men and 31,478 women) were included in the analysis.

This study was conducted in accordance with the Declaration of Helsinki, and the study protocol was approved by the ethics review board of all institutions and universities participating in the J-MICC Study.

### Medical examination data

We collected information on the results of medical examinations and complete-health checkups. In regions without linked medical examinations, medical examination items were measured independently. Medical examination items included height, weight, body mass index (BMI), systolic and diastolic blood pressures, serum levels of triglyceride and high-density lipoprotein cholesterol, fasting blood glucose level, HbA1c level, and other blood/biochemical test results.

Dyslipidemia was defined as a triglyceride level ≥150 mm Hg/dL, high-density lipoprotein cholesterol level <40 mg/dL, or the use of dyslipidemia medication. Hypertension was defined as systolic blood pressure ≥130 mm Hg, diastolic blood pressure ≥85 mm Hg, or the use of antihypertensive medication. Glucose intolerance was defined as a fasting blood glucose level ≥100 mg/dL, HbA1c level ≥5.6%, or the use of anti-diabetic medication. Obesity was defined as BMI ≥25 kg/m^2^.^21^

### Questionnaire surveys

Baseline surveys included a common questionnaire that collected information on sleep, exercise, alcohol drinking habits, smoking habits, psychological stress, use of medications and supplements, dietary habits (including food intake), and medical histories including those of family members (and a reproductive history in women).

To assess awareness of limiting food intake, participants were asked whether they avoid consumption of energy, fat, or sweets, with “yes” or “no” as responses. Those who answered “yes” were deemed to have awareness, indicating the subjective recognition for the restriction of food intake, rather than actual food restriction.

Furthermore, those who indicated that they have a habit of drinking alcoholic beverages at least once a month were regarded as “current drinkers,” and those who indicated that they were currently smoking were regarded as “current smokers.” The amount of habitual exercise was estimated by a method similar to the International Physical Activity Questionnaire (IPAQ).^22^ Habitual exercise was classified into three categories and assigned an exercise intensity as follows: “walking”, 3.3 metabolic equivalents of task (METs); “moderate activity”, 4.0 METs; and “vigorous activity”, 8.0 METs. MET values were calculated by multiplying the assigned intensity by the frequency and duration of each category. Additionally, daily activities were quantified by multiplying the duration of “force work,” “walking,” “standing,” and “sitting” with respective activity intensity values of 4.5, 3.0, 2.0, and 1.5 METs.^23^ The participants were divided into tertiles according the distribution of habitual exercise and daily activity.

### Energy and nutrient intake

Daily intake of energy (kcal) and fat (gram) was estimated using the Food Frequency Questionnaire (FFQ). Briefly, information on the dietary habits of the past year was collected, including the frequency of intake of 47 items (staples, foods, and beverages) and the amount of staple foods consumed for breakfast, lunch, and dinner. Estimated values for energy and fat intake on the FFQ have been validated using weighted diet records.^24^^–^^26^ Validity indices for energy estimates in males and females were reported as 0.40 and 0.44, respectively, and those for fat were reported as 0.62 and 0.48, respectively.^24^^–^^26^ For sweet foods, only frequency information was collected in the FFQ; accordingly, sugar intake could not be evaluated as a nutrient. Therefore, in the current study, sweet food was defined as cake and Japanese cake. The frequency of intake of cake and Japanese cake, beef and pork, green and yellow vegetables, and fruits were calculated as weekly averages, based on an 8-point scale (almost never eat, 1–3 times per month, 1–2 times per week, 3–4 times per week, 5–6 times per week, once per day, twice per day, and ≥3 times per day). Tertiles were created for each intake of beef and pork, green and yellow vegetables, and fruits for men and women and were used for statistical analysis.

### Follow-up and mortality data

Participants were followed up from the start of baseline survey, and the final year of the follow-up varied from the end of 2017 to the end of 2020, depending on the study area. Participants who moved out of study regions were censored. The duration of follow-up was calculated as the time from the date of the participant’s baseline survey to their death, move out of study regions, or end of the follow-up, whichever came first. During an average follow-up of 11 years (range: 0–15.9 years), 2,516 people died, and 3,154 people moved out of study regions. The information on death was confirmed using death certificates at the applicable health center, with the permission of the Japanese Ministry of Health, Labour and Welfare.

### Statistical analysis

The associations between awareness of limiting food intake and nutritional intake estimated using the FFQ were determined according to sex using multivariable regression analyses. Age, BMI, region, smoking and alcohol drinking habits, years of education, daily activity, and habitual exercise were used as covariates. In the analyses for the association with fat intake, the effect of estimated energy intake was additionally adjusted.

The distributions of age, BMI, and awareness of limiting food intake, but excluding that used as a dependent variable, were compared by awareness of limiting food intake using logistic regression models, and age was always included in the model (eTable 1, eTable 2, and eTable 3).

Cox proportional hazards modeling was used to evaluate the association between awareness of limiting food intake and mortality 1 year after the baseline survey; the hazard ratio (HR) and 95% confidence interval (CI) were calculated by sex. To infer causal relationships, we selected the covariates for the multivariate analysis based on lifestyle-related factors pertaining to metabolic syndrome diagnostic criteria and factors that would affect the association between awareness of limiting food intake and all-cause mortality, and these covariates were evaluated through drawing Direct Acyclic Graphs (DAG) (DAGitty3.0, http://www.dagitty.net/), and confirmed the effect by adjustment (total effect) for causal effect identification. The following factors were applied in the DAG: age (35–49, 50–59, and 60–69 years), BMI (<18.5, 18.5–24.9, and ≥25.0 kg/m^2^), 11 study regions, smoking status (current, past, and never), alcohol drinking habit (current, past, and never), years of education (<16 and ≥16 years), daily activity (tertile), habitual exercise (tertile), beef and pork intake (tertile), green and yellow vegetable intake (tertile), fruit intake (tertile), awareness of energy intake, awareness of limiting fat intake, awareness of limiting sweets intake, energy intake (continuous variable), fat intake (continuous variable), sweets intake (the more frequent intake value of either cake or Japanese cake), and the presence of dyslipidemia, hypertension, and glucose intolerance. For statistical models, we used variables that did not have a biasing path in the DAG (eFigure 1, eFigure 2, and eFigure 3).

The main causes of death in the study population were cancer and cerebrovascular disease, and metabolic syndrome is an important high-risk condition for these diseases. In general, individuals with metabolic syndrome are likely to have greater awareness of limiting food intake because of the need to manage these underlying diseases. To exclude the effects of causal reversals, subclass analyses were performed with stratification by referring to diagnostic criteria for metabolic syndrome: central obesity, dyslipidemia, hypertension, and hyperglycemia. Awareness of limiting energy intake was stratified by BMI, fat intake was stratified by dyslipidemia and BMI, and sweets intake was stratified by glucose intolerance and BMI.

In addition, we conducted a mediation analysis using the four-way effect decomposition to evaluate the association between fat intake, as a mediator of awareness of limiting fat intake, and all-cause mortality. This analysis can estimate the four-way decomposition of controlled direct effect, reference interaction (only interaction), mediated interaction, and pure indirect effect (only mediation). The exposure was awareness of limiting fat intake, and the mediator was fat intake (continuous variable). The average value of fat intake without awareness of limiting fat intake was set as a counterfactual mediator. We used a linear regression model to analyze the association with the mediator.^27^ We represented the sum of the effects of controlled direct effect and reference interaction as direct effect, and the sum of the effects of mediated interaction and pure indirect effect as indirect effect.

All statistical analyses were performed using Stata software version 17 (Stata Corp, College Station, TX, USA). The statistical significance level was set at 5%.

## RESULTS

Sex differences for each variable were evaluated using χ^2^-test for categorical variables and *t*-test for continuous variables, and the proportion of participants in the age group of 60–69 years was the highest for both men and women (Table 1). The prevalence of current smoker, current alcohol drinker, obesity, hypertension, impaired glucose tolerance, and dyslipidemia were higher in men than in women. In addition, women tended to show higher prevalence in the awareness of limiting each food intake; there were statistically significant differences between men and women, except for awareness of limiting sweets intake.

**Table 1.** Characteristics of the study population according to sex

	Men *N* = 27,294		Women *N* = 31,478		*P*
	*N (%)*				
Age, years					
35–49	6,383	(23.4)	8,057	(25.6)	*<0.001* ^a^
50–59	8,993	(33.0)	11,031	(35.0)	
60–69	11,918	(43.7)	12,390	(39.4)	
Years of education (≥16 years)	7,644	(36.1)	2,486	(10.9)	*<0.001* ^a^
Current smoker	8,201	(30.1)	2,032	(6.5)	*<0.001* ^a^
Current alcohol drinker	20,897	(76.6)	11,266	(35.8)	*<0.001* ^a^
Obese (BMI ≥25.0 kg/m^2^)	8,236	(30.2)	5,922	(18.8)	*<0.001* ^a^
Daily activity (≥15.0 METs·h/day)	8,637	(31.7)	9,506	(30.3)	*<0.001* ^a^
Habitual exercise (≥2.19 METs·h/day)	8,126	(30.7)	8,136	(27.0)	*<0.001* ^a^
Hypertension	16,577	(60.7)	14,684	(46.7)	*<0.001* ^a^
Glucose intolerance	8,299	(30.4)	6,617	(21.0)	*<0.001* ^a^
Dyslipidemia	11,555	(42.3)	8,638	(27.4)	*<0.001* ^a^
Food intake					
Beef and pork (≥3 times/week)	7,709	(28.3)	13,318	(42.4)	*<0.001* ^a^
Green & yellow vegetable (≥3 times/week)	11,553	(42.4)	17,873	(56.9)	*<0.001* ^a^
Fruits (≥3 times/week)	6,763	(24.8)	13,910	(44.2)	*<0.001* ^a^
Cake (≥1 time/week)	4,939	(18.3)	8,506	(27.3)	*<0.001* ^a^
Japanese cake (≥1 times/week)	7,447	(27.3)	15,204	(48.3)	*<0.001* ^a^
Awareness of limiting food intake					
Energy	9,219	(33.8)	12,379	(39.3)	*<0.001* ^a^
Fat	10,306	(37.8)	14,485	(46.0)	*<0.001* ^a^
Sweets	9,176	(33.6)	10,758	(34.2)	*0.155* ^a^
Three awareness (Yes) responses	6,567	(24.1)	8,679	(27.6)	*<0.001* ^a^
One to three awareness (Yes) responses	12,775	(46.8)	16,438	(52.2)	*<0.001* ^a^
	Mean (SD)				
Nutritional intake					
Energy, kcal/day	1,939.0	(356.1)	1,553.7	(230.6)	*<0.001* ^b^
Fat, g/day	42.6	(11.0)	44.8	(10.8)	*<0.001* ^b^

BMI, body mass index; MET, metabolic equivalent of task; SD, standard deviation.

^a^*P* values obtained using χ^2^ test.

^b^*P* values obtained using *t*-test.

The distributions of age, BMI, and awareness of limiting intake of fat, and sweets, were statistically different between groups with and without awareness of limiting energy intake (eTable 1). For the comparison between groups by the awareness of limiting fat intake, the distributions of all variables were significantly different (eTable 2). Similar analyses were conducted for awareness of limiting sweets intake. All variables shown in the tables were significantly related to awareness (eTable 3).

For both men and women, study participants with awareness of limiting energy intake consumed lower FFQ-based estimated energy intake than those without this awareness; similarly, those with awareness of limiting fat intake showed lower fat intake than those without this awareness (Table 2). Furthermore, both men and women with awareness of limiting sweets intake consumed less energy and fat than those without this awareness, except for fat intake in women.

**Table 2.** Estimated daily intake by FFQ at baseline surveys according to awareness of limiting food intake

	Mean of estimated intake (95% CI)								

	Awareness of limiting energy intake			Awareness of limiting fat intake			Awareness of limiting sweets intake		
		
	No	Yes	*P*	No	Yes	*P*	No	Yes	*P*
Men									
Energy intake, kcal	1,959.4	1,899.0	<0.001^a^	1,955.2	1,912.3	<0.001^a^	1,951.1	1,915.1	<0.001^a^
	(1,954.1–1,964.8)	(1,892.2–1,905.8)		(1,949.7–1,960.7)	(1,905.8–1,918.8)		(1,945.9–1,956.4)	(1,908.1–1,922.0)	
Fat intake, g	42.7	42.3	0.791^b^	42.9	42.0	<0.001^b^	42.9	42.0	0.001^b^
	(42.6–42.9)	(42.1–42.5)		(42.8–43.1)	(41.8–42.2)		(42.7–43.1)	(41.7–42.2)	
Women									
Energy intake, kcal	1,568.9	1,530.3	<0.001^a^	1,565.7	1,539.6	<0.001^a^	1,564.2	1,533.5	<0.001^a^
	(1,565.5–1,572.2)	(1,526.4–1,534.3)		(1,562.2–1,569.3)	(1,536.0–1,543.2)		(1,561.0–1,567.4)	(1,529.3–1,537.6)	
Fat intake, g	44.9	44.6	0.025^b^	45.1	44.4	<0.001^b^	45.1	44.3	0.196^b^
	(44.8–45.1)	(44.4–44.8)		(45.0–45.3)	(44.2–44.5)		(44.9–45.2)	(44.1–44.5)	

BMI, body mass index; CI, confidence interval.

^a^Adjusted for age, BMI, region, smoking habit, alcohol drinking habit, years of education, daily activity, habitual exercise.

^b^Adjusted for age, BMI, region, smoking habit, alcohol drinking habit, years of education, daily activity, habitual exercise, energy intake.

We checked the biasing paths that affect the causal path between awareness of limiting food intake and all-cause mortality using DAGs and included the factors related to the biasing paths as covariates in the statistical model. In men, awareness of limiting energy intake was associated with a decreased mortality risk (HR 0.79; 95% CI, 0.71–0.88) in model 1 (adjusted for age only); in the subclass analysis by BMI, this result was significant for BMI <18.5 kg/m^2^ and BMI 18.5–24.9 kg/m^2^. However, these associations disappeared in model 2 (adjusted for lifestyle-related confounding factors, awareness of limiting intake of fat and sweets) (Table 3). In women, on the other hand, awareness of limiting energy intake was associated with an increased mortality risk (HR 1.39; 95% CI, 1.06–1.81) in model 2; in the subclass analysis, this association was stronger in those with BMI ≥25.0 kg/m^2^ (HR 1.93; 95% CI, 1.13–3.27).

**Table 3.** Association between awareness of limiting energy intake and all-cause mortality

	Events (*n*)	Person- years	Hazard ratio (95% CI)^*^	

			Model 1	Model 2
Men				
Awareness, no	1,243	196,503	1.00	1.00
Awareness, yes	444	93,725	0.79 (0.71–0.88)	0.89 (0.74–1.07)

BMI, kg/m^2^				
<18.5				
Awareness, no	91	6,374	1.00	1.00
Awareness, yes	8	1,157	0.47 (0.23–0.97)	0.51 (0.16–1.66)
18.5–24.9				
Awareness, no	808	133,360	1.00	1.00
Awareness, yes	281	61,249	0.79 (0.69–0.90)	0.84 (0.67–1.06)
≥25.0				
Awareness, no	344	56,770	1.00	1.00
Awareness, yes	155	31,319	0.89 (0.73–1.08)	0.99 (0.72–1.38)

Women				
Awareness, no	557	218,022	1.00	1.00
Awareness, yes	272	126,458	0.95 (0.82–1.10)	1.39 (1.06–1.81)

BMI, kg/m^2^				
<18.5				
Awareness, no	42	19,802	1.00	1.00
Awareness, yes	11	7,716	0.77 (0.39–1.52)	0.78 (0.25–2.42)
18.5–24.9				
Awareness, no	382	157,992	1.00	1.00
Awareness, yes	172	92,648	0.87 (0.72–1.04)	1.30 (0.94–1.80)
≥25.0				
Awareness, no	133	40,228	1.00	1.00
Awareness, yes	89	26,095	1.16 (0.88–1.53)	1.93 (1.13–3.27)

BMI, body mass index; CI, confidence interval; HR, hazard ratio.

^*^Hazard ratio due to the awareness of limiting energy intake.

Model 1: Adjusted for age.

Model 2: Adjusted for age; BMI; region; smoking habit; alcohol drinking habit; years of education; daily activity; habitual exercise; meat, green vegetable, and fruit intake; awareness of limiting fat and sweet food intake.

Although awareness of limiting fat intake was negatively associated with male mortality risk (HR 0.79; 95% CI, 0.72–0.88), this significant association disappeared in model 2 (adjusted for lifestyle-related confounding factors; awareness of limiting intake of energy and sweets; and the presence of dyslipidemia or hypertension) (Table 4). Similar tendencies were observed regardless of the presence of dyslipidemia, presence of dyslipidemia without medication, and BMI of 18.5–24.9 kg/m^2^. In women, awareness of limiting fat intake was significantly associated with a decreased mortality risk even after adjusting for all confounding variables (HR 0.73; 95% CI, 0.55–0.94) (model 2).

**Table 4.** Association between awareness of limiting fat intake and all-cause mortality

	Events (*n*)	Person- years	Hazard ratio (95% CI)^*^	

			Model 1	Model 2
Men				
Awareness, no	1,166	184,743	1.00	1.00
Awareness, yes	521	105,485	0.79 (0.72–0.88)	0.95 (0.79–1.14)

Dyslipidemia				
No				
Awareness, no	617	107,148	1.00	1.00
Awareness, yes	243	58,510	0.72 (0.62–0.84)	0.93 (0.72–1.20)
Yes				
Awareness, no	549	77,595	1.00	1.00
Awareness, yes	278	46,974	0.86 (0.74–0.99)	0.99 (0.76–1.28)
Among participants with dyslipidemia				
Medication, no				
Awareness, no	452	65,362	1.00	1.00
Awareness, yes	182	33,187	0.83 (0.70–0.99)	1.00 (0.74–1.35)
Medication, yes				
Awareness, no	97	12,234	1.00	1.00
Awareness, yes	96	13,788	0.97 (0.73–1.29)	0.85 (0.48–1.48)

BMI, kg/m^2^				
<18.5				
Awareness, no	85	5,888	1.00	1.00
Awareness, yes	14	1,643	0.60 (0.34–1.06)	1.04 (0.41–2.68)
18.5–24.9				
Awareness, no	756	124,990	1.00	1.00
Awareness, yes	333	69,619	0.80 (0.70–0.91)	0.97 (0.77–1.21)
≥25.0				
Awareness, no	325	53,866	1.00	1.00
Awareness, yes	174	34,223	0.87 (0.72–1.04)	0.92 (0.66–1.28)

Women				
Awareness, no	531	194,956	1.00	1.00
Awareness, yes	298	149,524	0.80 (0.69–0.92)	0.73 (0.55–0.94)

Dyslipidemia				
No				
Awareness, no	351	142,417	1.00	1.00
Awareness, yes	189	104,805	0.79 (0.66–0.95)	0.74 (0.54–1.03)
Yes				
Awareness, no	180	52,539	1.00	1.00
Awareness, yes	109	44,719	0.80 (0.63–1.02)	0.69 (0.42–1.12)
Among participants with dyslipidemia				
Medication, no				
Awareness, no	102	35,359	1.00	1.00
Awareness, yes	55	23,088	0.90 (0.64–1.25)	0.62 (0.32–1.18)
Medication, yes				
Awareness, no	78	17,181	1.00	1.00
Awareness, yes	54	21,631	0.69 (0.48–0.99)	0.60 (0.27–1.30)

BMI, kg/m^2^				
<18.5				
Awareness, no	38	17,435	1.00	1.00
Awareness, yes	15	10,082	0.77 (0.42–1.42)	0.73 (0.27–1.96)
18.5–24.9				
Awareness, no	359	141,284	1.00	1.00
Awareness, yes	195	109,356	0.76 (0.64–0.91)	0.76 (0.55–1.05)
≥25.0				
Awareness, no	134	36,237	1.00	1.00
Awareness, yes	88	30,086	0.88 (0.67–1.15)	0.62 (0.37–1.05)

BMI, body mass index; CI, confidence interval; HR, hazard ratio.

^*^Hazard ratio due to the awareness of limiting fat intake.

Model 1: Adjusted for age.

Model 2: Adjusted for age; BMI; region; smoking habit; alcohol drinking habit; years of education; daily activity; habitual exercise; meat, green vegetable, and fruit intake; awareness of limiting energy and sweet food intake; dyslipidemia and hypertension.

In the mediation analysis for women, the coefficients for direct and total effects of awareness of limiting fat intake on all-cause mortality were significant, at −0.27 (95% CI, −0.47 to −0.08) and −0.27 (95% CI, −0.46 to −0.07), respectively, after adjusting the effects of covariates used in Table 4. In contrast, the indirect effect was not statistically significant (Coefficient = 0.008; 95% CI, −0.001 to 0.016).

Awareness of limiting sweets intake was significantly associated with a decreased mortality risk among men (model 1 in Table 5). In the subclass analysis of model 1 among men, similar negative associations were observed in those without glucose intolerance and in those with glucose intolerance without medication. However, again, this association disappeared after adjusting for the effects of potential confounding factors (model 2 in Table 5). Similar results were observed among women without glucose intolerance and those with a BMI of 18.5–24.9 kg/m^2^. In men with glucose intolerance, awareness of limiting sweet intake was marginally related to the increase of all-cause mortality in model 2 (HR 1.29; 95% CI, 0.99–1.69).

**Table 5.** Association between awareness of limiting sweets intake and all-cause mortality

	Events (*n*)	Person- years	Hazard ratio (95% CI)^*^	

			Model 1	Model 2
Men				
Awareness, no	1,201	196,493	1.00	1.00
Awareness, yes	486	93,735	0.87 (0.78–0.97)	1.10 (0.92–1.31)

Glucose intolerance				
No				
Awareness, no	785	144,475	1.00	1.00
Awareness, yes	219	55,993	0.76 (0.65–0.88)	0.96 (0.75–1.22)
Yes				
Awareness, no	416	52,018	1.00	1.00
Awareness, yes	267	37,742	0.93 (0.80–1.09)	1.29 (0.99–1.69)
Among participants with glucose intolerance				
Medication, no				
Awareness, no	317	45,229	1.00	1.00
Awareness, yes	154	27,911	0.82 (0.68–1.00)	1.12 (0.82–1.54)
Medication, yes				
Awareness, no	99	6,789	1.00	1.00
Awareness, yes	113	9,831	0.91 (0.69–1.20)	1.43 (0.81–2.52)

BMI (kg/m^2^)				
<18.5				
Awareness, no	87	6,178	1.00	1.00
Awareness, yes	12	1,352	0.56 (0.31–1.04)	1.15 (0.45–2.90)
18.5–24.9				
Awareness, no	777	134,007	1.00	1.00
Awareness, yes	312	60,602	0.90 (0.78–1.02)	1.18 (0.95–1.47)
≥25.0				
Awareness, no	337	56,307	1.00	1.00
Awareness, yes	162	31,782	0.90 (0.75–1.09)	0.99 (0.72–1.37)

Women				
Awareness, no	585	233,237	1.00	1.00
Awareness, yes	244	111,243	0.90 (0.78–1.05)	0.94 (0.73–1.21)

Glucose intolerance				
No				
Awareness, no	426	185,536	1.00	1.00
Awareness, yes	137	81,850	0.76 (0.63–0.93)	0.85 (0.62–1.16)
Yes				
Awareness, no	159	47,702	1.00	1.00
Awareness, yes	107	29,393	1.16 (0.90–1.49)	1.12 (0.72–1.73)
Among participants with glucose intolerance				
Medication, no				
Awareness, no	133	44,461	1.00	1.00
Awareness, yes	77	24,560	1.10 (0.83–1.47)	1.00 (0.62–1.60)
Medication, yes				
Awareness, no	26	3,241	1.00	1.00
Awareness, yes	30	4,833	0.93 (0.54–1.60)	1.31 (0.43–4.06)

BMI (kg/m^2^)				
<18.5				
Awareness, no	41	20,917	1.00	1.00
Awareness, yes	12	6,600	1.00 (0.52–1.92)	1.61 (0.52–4.98)
18.5–24.9				
Awareness, no	405	170,751	1.00	1.00
Awareness, yes	149	79,889	0.80 (0.67–0.97)	0.84 (0.62–1.15)
≥25.0				
Awareness, no	139	41,569	1.00	1.00
Awareness, yes	83	24,754	1.08 (0.82–1.43)	1.09 (0.67–1.79)

BMI, body mass index; CI, confidence interval; HR, hazard ratio.

^*^Hazard ratio due to the awareness of limiting sweets intake.

Model 1: Adjusted for age.

Model 2: Adjusted for age; BMI; region; smoking habit; alcohol drinking habit; years of education; daily activity; habitual exercise; meat, green vegetable, and fruit intake; awareness of limiting energy and fat intake; and glucose intolerance.

## DISCUSSION

This study evaluated the association between awareness of limiting food intake and all-cause mortality in the general Japanese population. Significant negative associations between awareness of limiting fat intake and mortality were observed in women. Mediation analysis revealed that this association was not mediated by actual fat intake. On the other hand, awareness of limiting energy intake was associated with an increased mortality risk in women, and this association was stronger in those with BMI ≥25.0 kg/m^2^.

Response to the questionnaire regarding awareness of limiting food intake was subjective in nature; as such, positive responses were not necessarily accompanied by actual restrictions in dietary behavior. Therefore, we conducted a mediation analysis to determine whether awareness of limiting fat intake led to lower mortality via actual fat intake reduction. The results of the mediation analysis showed that awareness of limiting fat intake, rather than actual reduction in fat intake, was significantly associated with lower all-cause mortality, especially among women. These results suggest that individuals with higher dietary awareness may have higher overall health awareness and healthier behaviors beyond dietary behaviors, and that this may be associated with lower all-cause mortality. This trend was more pronounced among women.

Health consciousness and related behaviors are not always in accordance. For example, it has been reported that the self-reported consumption of alcohol is underestimated.^28^ Furthermore, self-reported smoking rates tend to be underestimated, based on a literature review.^29^ In contrast, the amount of exercise is reported as overestimated.^30^ Further, self-reported food intake does not necessarily match the actual intake.^31^ The behavioral change stage model has five stages; precontemplation, contemplation, preparation, action, and maintenance; the stage with healthy awareness but without healthy behavior corresponds to a period of contemplation or preparation this model.^5^ As detailed in the introduction, campaigns in various countries have targeted awareness to promote healthy behavioral changes. Although studies suggest the success of these campaigns in increasing awareness and improving behavior, to the best of our knowledge, no study has evaluated the association between awareness of limiting food intake and mortality risk.

### Energy intake

In the present study, the estimated energy intake using FFQ was lower in those with awareness of limiting energy intake than in those without this awareness. However, in model 2, women with awareness of limiting energy intake showed an increased mortality risk (HR 1.39; 95% CI, 1.06–1.81), especially in those with BMI ≥25.0 kg/m^2^ (HR 1.93; 95% CI, 1.13–3.27). These inconsistent results might be due to a causal reversal phenomenon, in which participants with background risk factors for excessive energy intake (eg, high BMI) at the time of the baseline survey had energy intake restriction awareness, resulting in the observed increased mortality risk. To confirm this possibility, we re-conducted the same analyses after excluding the participants with either hypertension, dyslipidemia, or hyperglycemia at baseline surveys, and the results were almost same, except that the estimate of HR for obese women with BMI ≥25.0 kg/m^2^ was much higher, at 4.37 (95% CI, 1.06–18.03). Detailed analysis including data by cause of death is needed in the future.

### Fat intake

Fat intake has been reported to have a linear positive or U-shaped association with mortality.^3^^,^^32^ Regarding the association between awareness and behavior pertaining to fat intake, a previous study reported that subjective and objective assessments of fat intake did not match in both evaluated samples, reflecting the general population in the Netherlands and adults in the United States.^33^ In addition, it has been reported that fat intake, as well as energy intake, is reduced by food labeling.^13^

In the present study, the estimated fat intake using the FFQ was lower in those with awareness of limiting fat intake than in those without this awareness. Although no significant association was found between awareness of limiting fat intake and all-cause mortality in men (model 2), a significant negative association was observed among women (HR 0.73; 95% CI, 0.55–0.94 in model 2). Moreover, a similar (although nonsignificant) negative association was observed in women with obesity (HR 0.62; 95% CI, 0.37–1.05 in model 2). The mediation analysis revealed that these associations were not significantly mediated by actual fat intake, while significant negative associations were found for the direct and total effects for awareness of limiting fat intake on mortality risk.

Although a significant bias could occur if those with awareness of limiting food intake responded to the FFQ more conservatively than their actual intake, the results of the mediation analysis indicate that the effect via fat intake obtained from the FFQ was not significant. In other words, even if participants indicated a lower fat intake on the FFQ than their actual fat intake, other mechanisms might be responsible for the decline in all-cause mortality.

### Sweet food intake

In previous studies, excessive intake of added sugar^34^ and total sugar were associated with increased mortality risk.^4^ In contrast, another study reported no significant association between eating patterns for sweet foods and mortality.^35^

The current study did not find a significant association between awareness of limiting sweets intake and a decrease in all-cause mortality risk. There are two potential explanations for this result. First, it may be due to the infrequency of eating sweet foods relative to the energy and fat intake in the daily diet; as a result, the intake of sweet foods may have less impact on mortality. In fact, the percentage of those who consumed cakes and Japanese cake daily was quite small in the current study, comprising 0.2% of those with awareness of limiting sweet foods and 0.1% of those without this awareness. Second, we only had information on the frequency of sweet food intake, disallowing a detailed quantitative assessment and mediation analysis. Since a lot of sugar is consumed from foods other than cakes, such as sweets, breads, and soft drinks, future studies should take this consumption into account as well.

Only men with glucose intolerance showed a marginally significant positive association between awareness of limiting sweets intake and all-cause mortality in model 2 (HR 1.29; 95% CI, 0.99–1.69). This trend was enhanced among the participants with medication. Although a more detailed analysis is needed, these results suggest that there may be residual effects of causal reversal in the relationship between awareness of limiting sweet foods and all-cause mortality risk in men with impaired glucose tolerance.

### Strengths and limitations

To the best of our knowledge, this is the first study to examine associations between awareness of limiting food intake and the risk of mortality in a relatively large number of participants from the general population.

However, as a limitation of the present study, although this was a prospective study, the age at baseline was 35–69 years. Some participants already had a condition requiring dietary restrictions at baseline, which may have contaminated the results (eg, resulting in causal reversal). Therefore, we performed subclass analyses, excluding populations with underlying diseases requiring dietary restrictions. Furthermore, we adjusted for confounding factors using information on a wide range of lifestyle factors and medical examination results; however, the effects of host factors and unspecified confounding factors are unknown. Additionally, the results did not change even when categories were further divided. Furthermore, the present study targeted participants who underwent medical examinations and voluntary responded to mailed fliers. Accordingly, the proportion of participants with high health consciousness may be higher than that in the general population, and the results may be slightly overestimated.

Awareness of limiting food intake might be influenced by a history of disease and other factors. Subjective stress was considered a potential confounding factor but adjusting for simple subjective stress status at baseline (having experienced strong stress in the past year or not) did not affect the main results. We attempted to distinguish the effects of underlying health conditions from those of awareness using subclass analyses; however, we could not adjust for the effects of other unknown factors. Moreover, some participants may have been dieting, which is a potential confounding factor, but this information was not available.

In this study, actual fat intake was used as the most likely mediator in the mediation analysis of the awareness of limiting fat intake. However, since the study design was a cross-sectional study and the temporal order of causes and mediators was not ensured, it may not have been sufficiently assessed as a mediator, which is one of the limitations of this study.

Sugar intake was not evaluated as a nutrient since only frequency information for cake and Japanese cake was collected using the FFQ used in this study. Lastly, we could not consider salt intake in this study because of the low validity of salt intake using FFQ.

### Conclusions

This study examined the association between awareness of limiting food intake and all-cause mortality in the Japanese general population. Awareness of limiting fat intake was associated with lower risk of all-cause mortality only in women, and this association was not mediated by actual fat intake. On the other hand, awareness of limiting intake of energy and sweets did not reduce the risk of all-cause mortality. These results suggest that awareness of limiting food intake has a limited effect on all-cause mortality risk, and this relationship may reflect not only dietary habits, but also other behavioral changes and overall health awareness.

## SUPPLEMENTARY MATERIAL

eTable 1. Characteristics of the study population according to awareness of limiting energy intake

eTable 2. Characteristics of the study population according to awareness of limiting fat intake

eTable 3. Characteristics of the study population according to awareness of limiting sweets intake

eFigure 1. Directed acyclic graph of the association between awareness of limiting energy intake and all-cause mortality

eFigure 2. Directed acyclic graph of the association between awareness of limiting fat intake and all-cause mortality

eFigure 3. Directed acyclic graph of the association between awareness of limiting sweets intake and all-cause mortality

## References

[1] GBD 2017 Diet Collaborators. Health effects of dietary risks in 195 countries, 1990–2017: a systematic analysis for the Global Burden of Disease Study 2017. Lancet. 2019;393:1958–1972. 10.1016/S0140-6736(19)30041-8 30954305 PMC6899507

[2] Nagai M, Ohkubo T, Miura K, ; NIPPON DATA80 Research Group. Association of total energy intake with 29-year mortality in the Japanese: NIPPON DATA80. J Atheroscler Thromb. 2016;23:339–354. 10.5551/jat.29991 26460380

[3] Kang M, Park SY, Boushey CJ, . Does incorporating gender differences into quantifying a food frequency questionnaire influence the association of total energy intake with all-cause and cause-specific mortality? Nutrients. 2020;12:2914. 10.3390/nu12102914 32977670 PMC7598663

[4] Anderson JJ, Gray SR, Welsh P, . The associations of sugar-sweetened, artificially sweetened and naturally sweet juices with all-cause mortality in 198,285 UK Biobank participants: a prospective cohort study. BMC Med. 2020;18:97. 10.1186/s12916-020-01554-5 32326961 PMC7181499

[5] Prochaska JO, Velicer WF. The transtheoretical model of health behavior change. Am J Health Promot. 1997;12:38–48. 10.4278/0890-1171-12.1.38 10170434

[6] Rios LE, Herval ÁM, Ferreira RC, Freire M. Prevalences of stages of change for smoking cessation in adolescents and associated factors: systematic review and meta-analysis. J Adolesc Health. 2019;64:149–157. 10.1016/j.jadohealth.2018.09.005 30447952

[7] Robinson LM, Vail SR. An integrative review of adolescent smoking cessation using the Transtheoretical Model of Change. J Pediatr Health Care. 2012;26:336–345. 10.1016/j.pedhc.2010.12.001 22920775

[8] Greene GW, Redding CA, Prochaska JO, . Baseline transtheoretical and dietary behavioral predictors of dietary fat moderation over 12 and 24 months. Eat Behav. 2013;14:255–262. 10.1016/j.eatbeh.2013.01.014 23910762 PMC4008122

[9] Takeshima T, Okayama M, Harada M, Ae R, Kajii E. Effects of disclosing hypothetical genetic test results for salt sensitivity on salt restriction behavior. Int J Gen Med. 2013;6:361–368. 10.2147/IJGM.S44979 23696713 PMC3658438

[10] Marshall SJ, Biddle SJ. The transtheoretical model of behavior change: a meta-analysis of applications to physical activity and exercise. Ann Behav Med. 2001;23:229–246. 10.1207/S15324796ABM2304_2 11761340

[11] Lipschitz JM, Yusufov M, Paiva A, . Transtheoretical principles and processes for adopting physical activity: a longitudinal 24-month comparison of maintainers, relapsers, and nonchangers. J Sport Exerc Psychol. 2015;37:592–606. 10.1123/jsep.2014-0329 26866767

[12] Liu J, Mozaffarian D, Sy S, ; FOOD-PRICE (Policy Review and Intervention Cost-Effectiveness) Project. Health and economic impacts of the National Menu Calorie Labeling Law in the United States: a microsimulation study. Circ Cardiovasc Qual Outcomes. 2020;13:e006313. 10.1161/CIRCOUTCOMES.119.006313 32493057 PMC7299757

[13] Shangguan S, Afshin A, Shulkin M, ; Food PRICE (Policy Review and Intervention Cost-Effectiveness) Project. A meta-analysis of food labeling effects on consumer diet behaviors and industry practices. Am J Prev Med. 2019;56:300–314. 10.1016/j.amepre.2018.09.024 30573335 PMC6340779

[14] von Philipsborn P, Stratil JM, Burns J, . Environmental interventions to reduce the consumption of sugar-sweetened beverages and their effects on health. Cochrane Database Syst Rev. 2019;6:CD012292. 10.1002/14651858.CD012292.pub2 31194900 PMC6564085

[15] An R, Zheng J, Xiang X. Projecting the influence of sugar-sweetened beverage warning labels and restaurant menu labeling regulations on energy intake, weight status, and health care expenditures in US adults: a microsimulation. J Acad Nutr Diet. 2022;122:334–344. 10.1016/j.jand.2021.05.006 34689957

[16] Takatsuji Y, Ishiguro A, Asayama K, ; NIPPON DATA90 Research Group. Exercise habits are associated with improved long-term mortality risks in the nationwide general Japanese population: a 20-year follow-up of the NIPPON DATA90 Study. Tohoku J Exp Med. 2020;252:253–262. 10.1620/tjem.252.253 33162455

[17] Sakata R, McGale P, Grant EJ, Ozasa K, Peto R, Darby SC. Impact of smoking on mortality and life expectancy in Japanese smokers: a prospective cohort study. BMJ. 2012;345:e7093. 10.1136/bmj.e7093 23100333 PMC3481021

[18] Hamajima N; J-MICC Study Group. The Japan Multi-Institutional Collaborative Cohort Study (J-MICC Study) to detect gene-environment interactions for cancer. Asian Pac J Cancer Prev. 2007;8:317–323. 17696755

[19] Wakai K, Hamajima N, Okada R, ; J-MICC Study Group. Profile of participants and genotype distributions of 108 polymorphisms in a cross-sectional study of associations of genotypes with lifestyle and clinical factors: a project in the Japan Multi-Institutional Collaborative Cohort (J-MICC) Study. J Epidemiol. 2011;21:223–235. 10.2188/jea.JE20100139 21467728 PMC3899413

[20] Takeuchi K, Naito M, Kawai S, . Study profile of the Japan Multi-institutional Collaborative Cohort (J-MICC) Study. J Epidemiol. 2021;31:660–668. 10.2188/jea.JE20200147 32963210 PMC8593573

[21] Yamagishi K, Iso H. The criteria for metabolic syndrome and the national health screening and education system in Japan. Epidemiol Health. 2017;39:e2017003. 10.4178/epih.e2017003 28092931 PMC5343105

[22] Craig CL, Marshall AL, Sjöström M, . International physical activity questionnaire: 12-country reliability and validity. Med Sci Sports Exerc. 2003;35:1381–1395. 10.1249/01.MSS.0000078924.61453.FB 12900694

[23] America College of Sports Medicine. ACSM’S Metabolic Calculations Handbook. Lippencoctt Williams & Wilkins; 2006.

[24] Tokudome S, Goto C, Imaeda N, Tokudome Y, Ikeda M, Maki S. Development of a data-based short food frequency questionnaire for assessing nutrient intake by middle-aged Japanese. Asian Pac J Cancer Prev. 2004;5:40–43. 15075003

[25] Tokudome Y, Goto C, Imaeda N, . Relative validity of a short food frequency questionnaire for assessing nutrient intake versus three-day weighed diet records in middle-aged Japanese. J Epidemiol. 2005;15:135–145. 10.2188/jea.15.135 16141632 PMC7851066

[26] Imaeda N, Goto C, Sasakabe T, . Reproducibility and validity of food group intake in a short food frequency questionnaire for the middle-aged Japanese population. Environ Health Prev Med. 2021;26:28. 10.1186/s12199-021-00951-3 33653279 PMC7923820

[27] Discacciati A, Bellavia A, Lee JJ, Mazumdar M, Valeri L. Med4way: a Stata command to investigate mediating and interactive mechanisms using the four-way effect decomposition. Int J Epidemiol. 2018. 10.1093/ije/dyy236 30452641

[28] Stockwell T, Zhao J, Greenfield T, Li J, Livingston M, Meng Y. Estimating under- and over-reporting of drinking in national surveys of alcohol consumption: identification of consistent biases across four English-speaking countries. Addiction. 2016;111:1203–1213. 10.1111/add.13373 26948693 PMC4899158

[29] Connor Gorber S, Schofield-Hurwitz S, Hardt J, Levasseur G, Tremblay M. The accuracy of self-reported smoking: a systematic review of the relationship between self-reported and cotinine-assessed smoking status. Nicotine Tob Res. 2009;11:12–24. 10.1093/ntr/ntn010 19246437

[30] Ronda G, Van Assema P, Brug J. Stages of change, psychological factors and awareness of physical activity levels in The Netherlands. Health Promot Int. 2001;16:305–314. 10.1093/heapro/16.4.305 11733449

[31] Macdiarmid J, Blundell J. Assessing dietary intake: who, what and why of under-reporting. Nutr Res Rev. 1998;11:231–253. 10.1079/NRR19980017 19094249

[32] Kwon YJ, Lee HS, Park JY, Lee JW. Associating intake proportion of carbohydrate, fat, and protein with all-cause mortality in Korean adults. Nutrients. 2020;12:3208. 10.3390/nu12103208 33096652 PMC7589789

[33] Glanz K, Brug J, van Assema P. Are awareness of dietary fat intake and actual fat consumption associated?-a Dutch-American comparison. Eur J Clin Nutr. 1997;51:542–547. 10.1038/sj.ejcn.1600442 11248880

[34] Ramne S, Alves Dias J, González-Padilla E, . Association between added sugar intake and mortality is nonlinear and dependent on sugar source in 2 Swedish population-based prospective cohorts. Am J Clin Nutr. 2019;109:411–423. 10.1093/ajcn/nqy268 30590448

[35] Zupo R, Sardone R, Donghia R, . Traditional dietary patterns and risk of mortality in a longitudinal cohort of the Salus in Apulia Study. Nutrients. 2020;12:1070. 10.3390/nu12041070 32290631 PMC7230634

