# Supplementary material for: Association Between Awareness of Limiting Food Intake and All-cause Mortality: A Cohort Study in Japan
Source: J Epidemiol. 2024 Jun 5;34(6):286–94. doi: 10.2188/jea.JE20220354 (PMC11078597; doi:10.2188/jea.JE20220354)

eTable 1. Characteristics of the study population according to awareness of limiting energy intake

	Awareness of limiting energy intake				*P*
	No		Yes		
	N (%)				
Men	18,075	(100)	9,219	(100)	
Age, years					
35–49	4,249	(23.5)	2,134	(23.2)	
50–59	6,264	(34.7)	2,729	(29.6)	*<0.001*[a]
60–69	7,562	(41.8)	4,356	(47.3)	
Awareness of limiting food intake					
Fat intake, yes	2,479	(13.7)	7,827	(84.9)	*<0.001*[b]
Sweets intake, yes	2,084	(11.5)	7,092	(76.9)	*<0.001*[b]
BMI, kg/m^2					
<18.5	627	(3.5)	114	(1.2)	
18.5–24.9	12,293	(68.0)	6,024	(65.3)	*<0.001*[b]
≥25.0	5,155	(28.5)	3,081	(33.4)	
Women	19,099	(100)	12,379	(100)	
Age, years					
35–49	4,905	(25.7)	3,152	(25.5)	
50–59	6,999	(36.7)	4,032	(32.6)	*<0.001*[a]
60–69	7,195	(37.7)	5,195	(42.0)	
Awareness of limiting food intake					
Fat intake, yes	3,454	(18.1)	11,034	(89.1)	*<0.001*[b]
Sweets intake, yes	1,679	(8.8)	9,080	(73.3)	*<0.001*[b]
BMI, kg/m^2					
<18.5	1,852	(9.7)	811	(6.6)	
18.5–24.9	13,824	(72.4)	9,070	(73.3)	*<0.001*[b]
≥25.0	3,424	(17.9)	2,500	(20.2)	

BMI, body mass index.

[a]*P* values obtained by χ^2 test.

[b]*P* values obtained by logistic regression analysis after adjusted for age (a categorical variable).

eTable 2. Characteristics of the study population according to awareness of limiting fat intake

	Awareness of limiting fat intake				*P*
	No		Yes		
	N (%)				
Men	16,988	(100)	10,306	(100)	
Age, years					
35–49	4,252	(25.0)	2,131	(20.7)	
50–59	5,858	(34.5)	3,135	(30.4)	*<0.001*[a]
60–69	6,878	(40.5)	5,040	(48.9)	
Awareness of limiting food intake					
Energy intake, yes	1,392	(8.2)	7,827	(76.0)	*<0.001*[b]
Sweets intake, yes	1,602	(9.4)	7,574	(73.5)	*<0.001*[b]
Dyslipidemia					
Yes	6,972	(41.0)	4,583	(44.5)	*<0.001*[b]
Medication, yes	1,145	(16.4)	1,429	(31.2)	*<0.001*[b]
BMI, kg/m^2					
<18.5	578	(3.4)	163	(1.6)	
18.5–24.9	11,508	(67.7)	6,809	(66.1)	*<0.001*[b]
≥25.0	4,902	(28.9)	3,334	(32.4)	
Women	16,993	(100)	14,485	(100)	
Age, years					
35–49	4,635	(27.3)	3,422	(23.6)	
50–59	6,191	(36.4)	4,840	(33.4)	*<0.001*[a]
60–69	6,167	(36.3)	6,223	(43.0)	
Awareness of limiting food intake					
Energy intake, yes	1,347	(7.9)	11,032	(76.2)	*<0.001*[b]
Sweets intake, yes	1,006	(5.9)	9,752	(67.3)	*<0.001*[b]
Dyslipidemia					
Yes	4,352	(25.6)	4,286	(29.6)	*<0.001*[b]
Medication, yes	1,435	(33.0)	2,169	(50.6)	*<0.001*[b]
BMI, kg/m^2					
<18.5	1,616	(9.5)	1,047	(7.2)	
18.5–24.9	12,293	(72.3)	10,600	(73.2)	*<0.001*[b]
≥25.0	3,084	(18.2)	2,838	(19.6)	

BMI, body mass index.

[a]*P* values obtained by χ^2 test.

[b]*P* values obtained by logistic regression analysis after adjusted for age (a categorical variable).

eTable 3. Characteristics of the study population according to awareness of limiting sweets intake

	Awareness of limiting sweets intake				*P*
	No		Yes		
	N (%)				
Men	18,118	(100)	9,176	(100)	
Age, years					
35–49	4,483	(24.7)	1,900	(20.7)	
50–59	6,241	(34.5)	2,752	(30.0)	*<0.001*[a]
60–69	7,394	(40.8)	4,524	(49.3)	
Awareness of limiting food intake					
Energy intake, yes	2,127	(11.7)	7,092	(77.3)	*<0.001*[b]
Fat intake, yes	2,732	(15.1)	7,574	(82.5)	*<0.001*[b]
Glucose intolerance					
Yes	4,682	(25.8)	3,617	(39.4)	*<0.001*[b]
Medication, yes	620	(13.2)	1,013	(28.0)	*<0.001*[b]
BMI, kg/m^2					
<18.5	606	(3.3)	135	(1.5)	
18.5–24.9	12,363	(68.2)	5,954	(64.9)	*<0.001*[b]
≥25.0	5,149	(28.4)	3,087	(33.6)	
Women	20,720	(100)	10,758	(100)	
Age, years					
35–49	5,700	(27.5)	2,357	(21.9)	
50–59	7,539	(36.4)	3,492	(32.5)	*<0.001*[a]
60–69	7,481	(36.1)	4,909	(45.6)	
Awareness of limiting food intake					
Energy intake, yes	3,300	(15.9)	9,079	(84.4)	*<0.001*[b]
Fat intake, yes	4,733	(22.8)	9,752	(90.7)	*<0.001*[b]
Glucose intolerance					
Yes	3,938	(19.0)	2,679	(24.9)	*<0.001*[b]
Medication, yes	277	(7.0)	465	(17.4)	*<0.001*[b]
BMI, kg/m^2					
<18.5	1,975	(9.5)	688	(6.4)	
18.5–24.9	15,160	(73.2)	7,733	(71.9)	*<0.001*[b]
≥25.0	3,585	(17.3)	2,337	(21.7)	

BMI, body mass index.

[a]*P* values obtained by χ^2 test.

[b]*P* values obtained by logistic regression analysis after adjusted for age (a categorical variable).

eFigure 1. Directed acyclic graph of the association between awareness of limiting energy intake and all-cause mortality

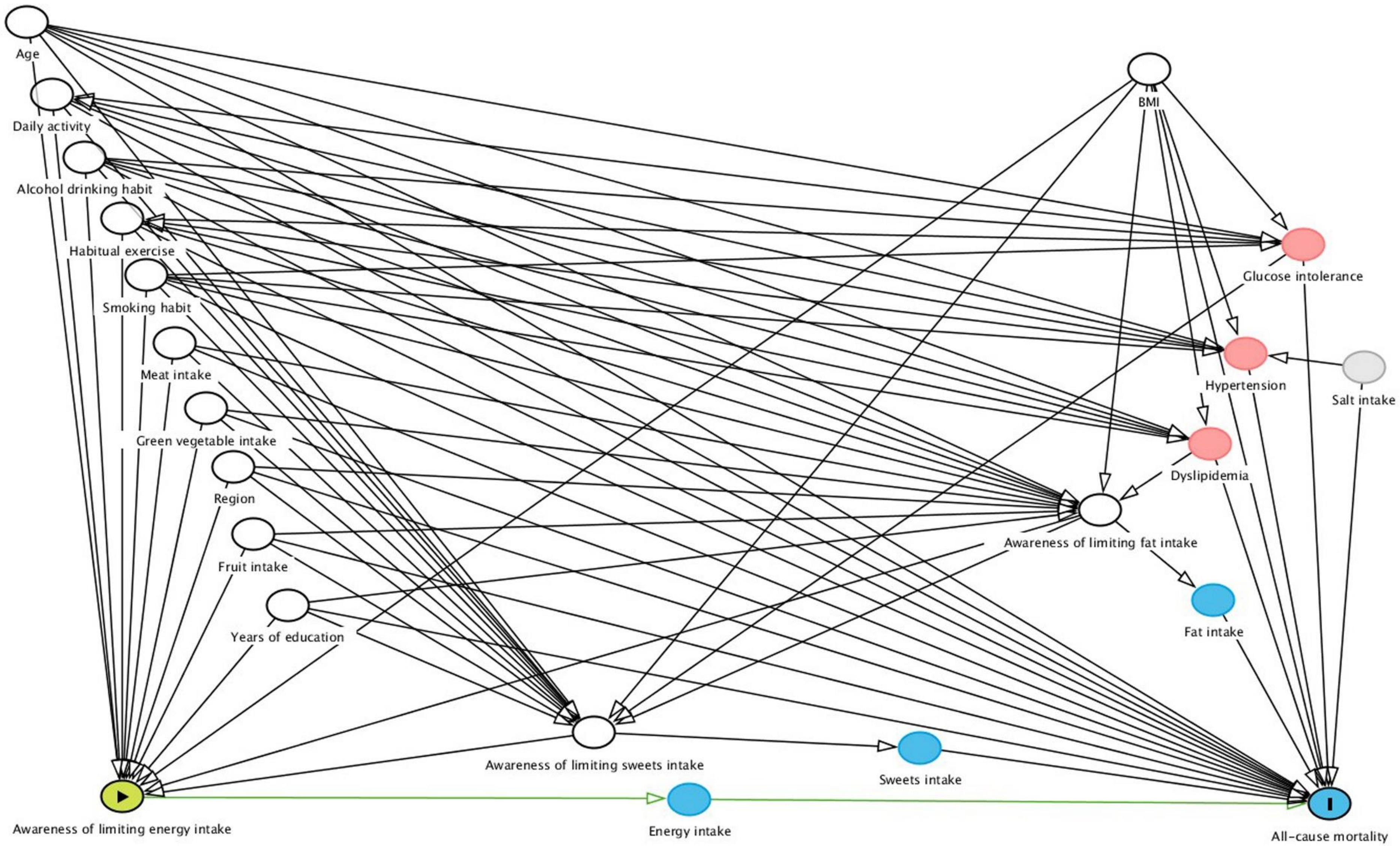

eFigure 2. Directed acyclic graph of the association between awareness of limiting fat intake and all-cause mortality

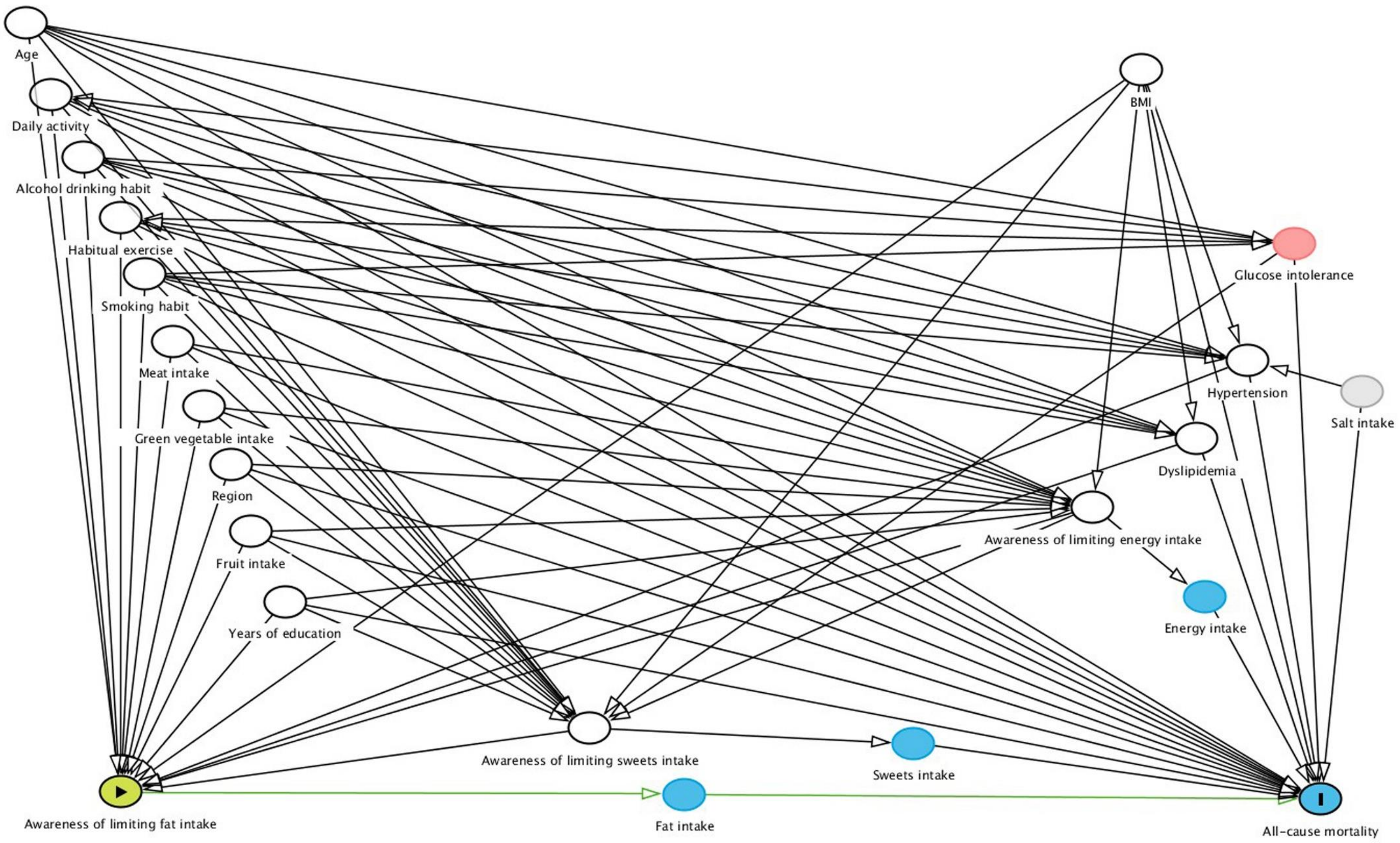

eFigure 3. Directed acyclic graph of the association between awareness of limiting sweets intake and all-cause mortality

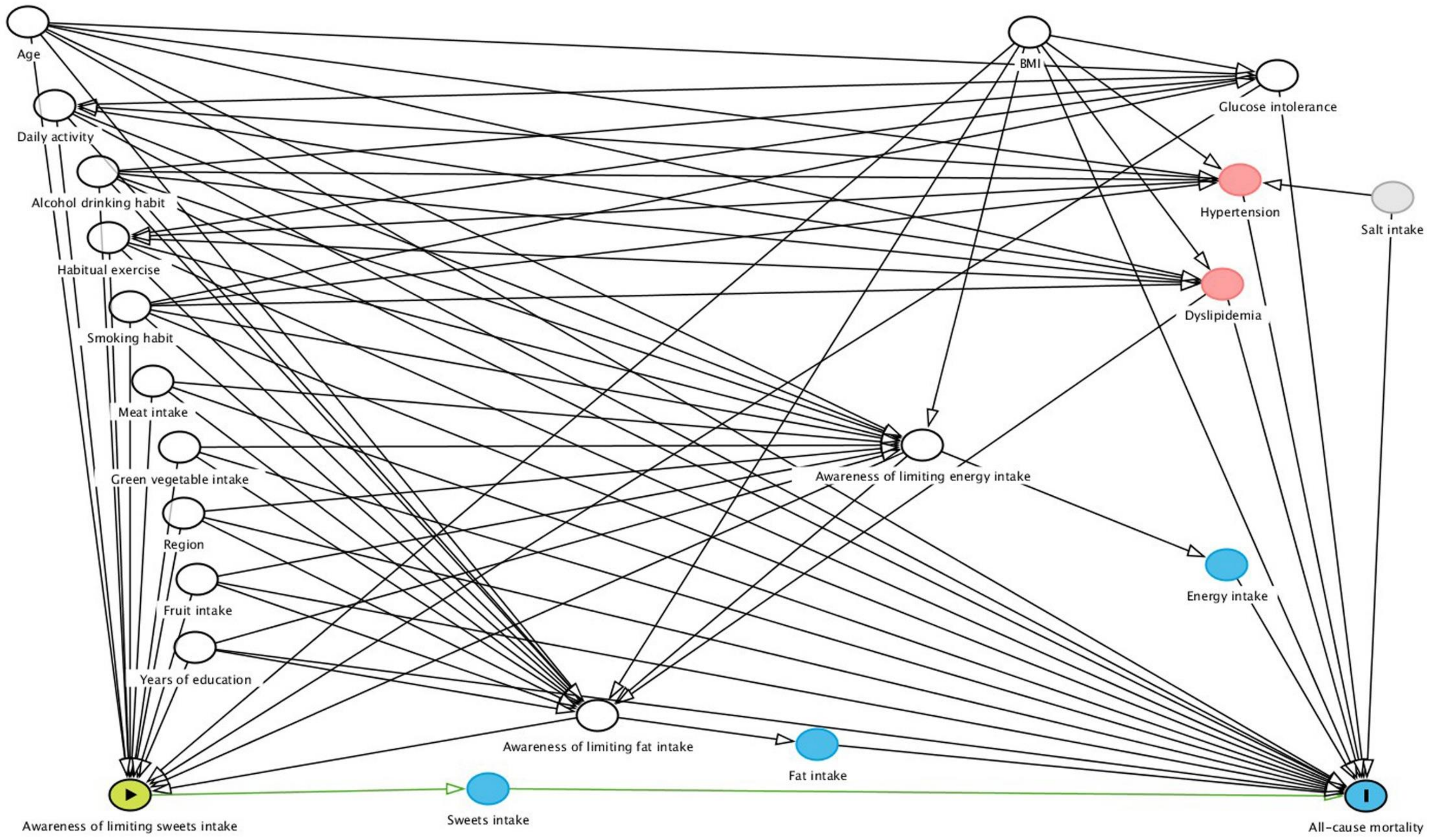

Supplement: Supplementary file 1 [file je-34-286-s001.pdf]
